# Do Chefs Value Health in Their Olive Oil Purchasing Decisions?

**DOI:** 10.3390/nu13020445

**Published:** 2021-01-29

**Authors:** María Gutierrez-Salcedo, Manuel Parras-Rosa, Francisco José Torres-Ruiz, Manuela Vega-Zamora

**Affiliations:** 1 Department of Business Management, Marketing and Sociology, Faculty of Social Sciences and Law, University of Jaén, 23071 Jaén, Spain; mparras@ujaen.es (M.P.-R.); ftorres@ujaen.es (F.J.T.-R.); mvega@ujaen.es (M.V.-Z.); 2 University Institute for Olive Grove and Olive Oil Research, University of Jaen, Campus las Lagunillas s/n, 23071 Jaén, Spain; 3 Agri-Food Campus of International Excellence (ceiA3), 14071 Córdoba, Spain

**Keywords:** olive oils, chefs, health, healthy foods, consumer behaviour

## Abstract

Amidst the public’s growing preoccupation with healthy eating, both inside and outside the home; an increase in people eating out; and the importance that olive oil has acquired in the markets due to its health benefits, the aim of this study is to find out whether health is also a relevant criterion for chefs in their olive oil purchase decisions. To this end, a survey was conducted of 400 chefs in Spain belonging to the international chefs’ association Euro-Toques. The results show that only 2% of the sample consider health to be a relevant criterion in the purchase of olive oils and that the attribute of “health” is not used by restaurants as an element of differentiation by which to position themselves in the market. These results lead us to conclude that the consumer demand for healthy foods (in this case, olive oils) is not being met by the restaurant sector. Moreover, this raises the question as to whether chefs actually make good opinion leaders, with all that this social role implies.

## 1. Introduction

Food scandals have shaken the world more than a few times in recent history (to name but a few, mad cow disease, bird flu, the listeria outbreak caused by larded meats, and even COVID-19). These, along with pesticide use in agriculture, antibiotics and hormones in cattle feed, and the spread of genetically modified crops have sharpened consumer awareness regarding nutrition, health, and food quality [1,2,3]. Thus, perceived food risks and a loss of confidence in the quality of conventional foods have led to a heightened interest in foods perceived to be healthier [2,3]. Perceiving that foods are healthy is a key determinant in their consumption [4,5,6]. Proof of this can be seen, for example, in the worldwide growth of the organic food market [7], as it is generally perceived to be a healthier food choice [8,9], or adherence to a Mediterranean diet for its beneficial health effects [10,11,12]. 

In this regard, olive oil, as its main source of fat, is the key ingredient in the Mediterranean diet [11,13]. Moreover, numerous studies provide evidence that olive oil, especially extra virgin olive oil (the highest quality), is a potent source of Vitamin E and polyphenols, and has strong antioxidant and antithrombotic action. It has also been shown that, in comparison to other oils (such as olive pomace oil, sunflower oil, or coconut oil), it reduces the risk of heart disease, diabetes, hypertension, cardiovascular disease-related mortality, and various forms of cancer [12,14,15,16,17,18,19,20,21,22,23]. 

Olive oil has therefore piqued the interest of consumers the world over [24]. In fact, worldwide olive oil demand has doubled in the last 30 years (1990–2020), rising from 1.6 to 3 million tonnes; although this phenomenon can be attributed to various factors, such as population growth, globalization, the opening up of markets, and even promotional campaigns to boost demand, one of the biggest determining factors is its proven health benefits [25,26,27]. This increase in consumption has even been noted in countries that produce very little olive oil or none at all, that do not traditionally follow a Mediterranean diet, such as Brazil, Canada, China, Japan, Switzerland, Mexico, and the US [28], where olive oil’s status as a healthy fat is touted as the main reason for its purchase and consumption [29,30]. Public awareness of health issues and olive oil quality is also growing in countries like Spain, the biggest olive oil producer in the world, where the consumption of extra virgin olive oil is on the rise [28], so there is a clear shift in demand towards higher quality and healthier olive oils.

In short, this consumer preoccupation with health in general, having transcended the “healthy lifestyle” trend [31], has now grown to become the main motivator for buyers or as the most highly valued attribute in the purchase of foods for consumption at home [32,33,34,35] and also when eating out [36,37,38].

Against this backdrop, Spanish consumers have started to eat out more regularly in restaurants and cafés in recent years [39]. Thus, in order for consumers to keep to a high quality and, above all, healthy diet, these establishments should be offering foods that fulfil these requirements. The key question, therefore, is: do establishments that provide food outside the home follow healthy eating guidelines in their food purchasing decisions? In other words, is health one of their main purchase criteria?

The initial hypothesis of this study is that food choice motives in the restaurant sector do not coincide with those of consumers, which poses a public health problem: the more you eat out, the less healthily you eat. 

The aim of this study is, therefore, to determine which attributes or factors drive the buying behaviour of chefs (on the premise that they are the decision makers in the restaurant kitchen), what they look for and value when buying olive oils, and what role the attribute of “health” plays within this group.

The results obtained show that only 2% of the restaurants interviewed cite health as an important factor in their olive oil buying decisions and that only 1.5% define their cuisine as “healthy”. These results are clearly relevant for consumers, who will realize that the food priorities and preferences of these establishments differ from their own; a factor of which they should be aware, as it directly affects their health (one of their principal concerns). Moreover, the results can help to steer the strategies of sector agents who, through market orientation, aim to sell their extra virgin olive oil to the restaurant and hospitality sector in general, and to fine dining restaurants in particular. Lastly, they are also important for government agencies that promote the use of olive oils in healthy eating campaigns.

The originality of this study lies in its focus on the restaurant sector, which serves to fill a gap in the specialized literature, given that the majority of studies on olive oil consumer behaviour and the value placed on the “health” attribute are mainly centred around the end consumer in the home environment [40,41,42,43,44,45,46,47].

## 2. Materials and Methods

### 2.1. Data Collection

The sample consisted of chefs working in Spain who currently use or have used olive oils in their restaurant kitchens and dining rooms. A total of 400 surveys were conducted by phone or onsite at the restaurant (all the responses of the sample were valid). The interviewees were selected from the Euro-Toques Association, which champions a healthy diet based on the use of quality products. Euro-Toques is an international chefs’ organization with a membership of more than 3500 chefs from 18 European countries, where Spain has 800 members (www.euro-toques.es). It aims to protect European culinary heritage in its diversity and its origins. The survey was administered by a market research company, which was instructed to ensure adequate representation in the sample of the whole range of restaurants belonging to the association. 

### 2.2. Measures

The survey questionnaire is divided into three sections (available as Supplementary Material published online). The first and second include questions on consumption of olive oils, and the selection criteria applied when purchasing them for cooking. The final section includes descriptors of the interviewee and the restaurant (such as age, gender, type of culinary training, type of cuisine offered, and the quality of the restaurant). Table 1 shows the profile of the final sample.

The starting point for our analysis was the assumption that usage and purchase factors vary depending on the context [29,48]. In this regard, we considered two clearly distinct settings: the dining room and kitchen. It makes sense to assume that the visibility of the oils and their containers in the former, and the recommendations made by serving staff to their customers (to give a good impression of service quality), can considerably affect the criteria used in buying decisions. In the case of the kitchen, therefore, it is logical to assume that other factors (price, health, flavour, or confidence in the supplier) will grow in importance relative to the visual signs that can be perceived by the consumer (packaging, brand, awards, etc.).

In the specific case of reasons for purchase, the questionnaire asks (for each type of oil) “What factors do you consider to be most important when buying extra virgin olive oil for your kitchen?”, and then asks the same question with regard to the dining room. It is important to note that the interviewee answered spontaneously rather than choosing from a predefined list of options. When the interviewee named a factor, they were prompted, “Is there anything else?”, until a maximum of 3 factors was reached. When the interviewee did not name any more, the same process was repeated with the next type of oil (virgin olive oil and olive oil).

### 2.3. Data Analysis

Although restaurants belonging to the Euro-Toques Association are subject to rules and guidelines on food quality and safety, in order to analyse the role of the “health” criterion in the olive oil purchase decisions of the chefs at the head of the selected restaurants, a restaurant quality scale was established ranging from band 1 (highest quality) to band 5 (lowest quality) based on the following criteria: (1) 2–3 Michelin stars or 3 Repsol suns; (2) 1 Michelin star or 2 Repsol suns; (3) 1 Repsol sun; (4) Average menu price > 30 € and (5) Average menu price < 30 €.

Continuous variables are described as means, and categorical variables are presented as percentages. The Mann–Whitney U test, chi-squared test, and ANOVA were used to analyse purchase criteria and to define the profile of the chefs that cite health among these criteria. 

Statistical analyses were performed using SPSS (SPSS for Windows, version 26.0, SPSS, Chicago, IL, USA). 

## 3. Results and Discussion

Table 2 details the factors, attributes, or reasons for purchase cited by the chefs interviewed, distinguishing between the purchase of oil for kitchen or dining room use, and between the three different types of olive oil (extra virgin, virgin. Or olive oil).

In general, as Table 2 shows, the distribution of the attributes or reasons for purchase is very similar, irrespective of whether the oil is for use in the kitchen or dining room; and even of the type of olive oil, which is a surprising result, considering the different functions and clear differences between the three oil types and the visibility of oils used in the dining room. Thus, regardless of type of oil and use, flavour is by far the most common purchase criterion or factor cited by the interviewees, followed by price, confidence in the supplier, area of origin, and olive variety. Overall, although price loses relative importance in the case of extra virgin olive oil (EVOO), the ranking of reasons and attributes is similar, both between types of oil and between uses.

From a negative standpoint, results worth noting include the low importance of internal attributes, such as organic production, health, and PDO, along with the little weight carried by an external attribute, such as brand.

In concrete terms, of the 400 chefs interviewed, only eight named the “health” attribute as a purchase criterion for olive oils, irrespective of usage location (kitchen or dining room), type of oil, and order of choice of the attribute. In other words, the health benefit of olive oils is not an attribute that drives the buying behaviour of chefs in Spain, even in the case of EVOO. This outcome clearly indicates that producers who seek to differentiate their olive oils from competitors by incorporating elements that bring greater health benefits to consumers will not find sales opportunities in the restaurant market, unlike in the home market. 

One important question is the homogeneity or heterogeneity of these attributes or buying reasons in relation to the restaurant quality scale. Restricting the analysis to EVOO, the healthiest olive oil, the potential association between citing each criterion or not and the quality level of the restaurant was established through a series of Mann–Whitney tests. The results show that there is only a weak association in the case of brand in the dining room (*p* = 0.016), in that it is slightly more important to lower-quality restaurants, and in the case of health in the dining room (*p* = 0.033), which is somewhat more important to higher-quality restaurants (Table 3). However, these small exceptions aside, the reality is that the profile of purchase criteria is very similar, both between kitchen and dining room, and across the different restaurant categories.

Despite the imbalance in the sample between chefs who use the “health” attribute as a purchase criterion (who we might call “healthy chefs”) and those who do not, the comparative analysis shows certain characteristics that differentiate the first group from the second.

**Figure 1:**
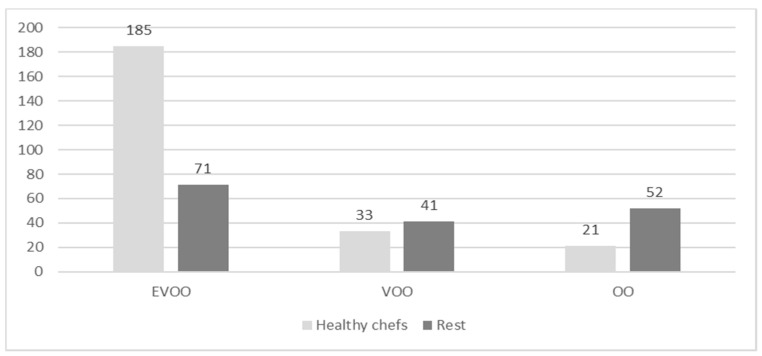
Average monthly consumption of olive oils in kitchen and dining room (litres). Extra virgin olive oil (EVOO), virgin olive oil (VOO), and olive oil (OO).

The ANOVA for the three oil qualities shows that the chefs who are most preoccupied with health are those that purchase and consume the most EVOO in their restaurants (F-test 6.415, *p* = 0.012). As Figure 1 indicates, EVOO consumption in this group is more than double that of chefs who do not use the “health” criterion.

On the other hand, it is important to point out that the chefs were asked in the questionnaire to describe their cuisine freely in their own terms. Only six of the 400 interviewed (1.5%) mentioned that their restaurant offered healthy food and, curiously, none of these chefs named the “health” attribute among their olive oil purchase criteria. If they do not take health into account when buying olive oil, can we really assume that they do so for other types of food? This behaviour would not be in line with that of an establishment that markets itself as a healthy option, in order to stand out from the competition.

With regard to the sociodemographic characteristics of the chefs that use the “health” attribute as a reason for purchase, it is worth noting that the variance analysis does not show significant differences in age or level of training. However, differences were observed in terms of gender (3.496, *p* = 0.062). Although overall there are more men in the sample than women, the percentage of women who use this criterion is higher (5.17%) than that of men (1.46%), a predictable result considering that women are more health conscious than men [49].

This leads us to conclude that our initial hypothesis is sound: chefs in Spain do not consider health to be among the main purchase criteria for olive oils, despite this being the main buying motive of consumers in their food choices, both inside and outside the home. The number of chefs from quality restaurants that apply the “health” criterion in their olive oil buying decisions is very low. Moreover, the “health” attribute is not used to set the restaurants apart, i.e., it is not used as an element of differentiation in the market.

Furthermore, in recent years, two population groups have been identified as the most influential EVOO opinion leaders in the world: health professionals (doctors, nutritionists, etc.) and chefs from famous restaurants. Chefs have accrued extraordinary kudos through their high media power and ability to influence the public in all food-related matters. Evidence of this can be seen in the glut of gastronomy and cookery shows on TV and the growing number of websites, video channels, and social media accounts providing information on foods and how to cook them. This growing consumer interest has turned chefs into influencers, and many have become the “face” of particular food products or brands [50]. However, the results of this study throw doubt onto whether they are, in general, deserving of the status of olive oil opinion leaders, given that they do not value health in their buying decisions. Nevertheless, it was also observed that those who do value health in their purchases buy more EVOO than those who do not. As a result, there is a positive correlation between increased demand of EVOO in the restaurant sector and placing value on the “health” attribute. 

Some lessons can be drawn from the above observations. Firstly, it may be of benefit to reformulate the strategy to boost demand in the restaurant sector implemented by entities such as the International Olive Council or the Spanish Olive Oil Interprofessional Organization. Indeed, many resources are already poured into communication campaigns aimed at chefs. However, these campaigns should go into more depth on the health benefits of consuming olive oils, with explicit messages [51] and health claims related to reducing disease risks, rather than functional claims [27]. The pairing of health professionals and chefs should be potentiated in these campaigns. In this regard, one project that stands out is the Torribera Mediterranean Center, founded by the Culinary Institute of America and the University of Barcelona, where chefs and scientists debate on which foods are considered to form part of the Mediterranean Diet in restaurants by analysing health and nutrition strategies. 

Secondly, we should reiterate the sequence: placing value on health—increase in EVOO consumption in the restaurant sector—increase in EVOO demand. Boosting EVOO demand in the restaurant distribution channel, together with increasing EVOO consumption among young people, is a priority strategy of the Spanish public authorities and of the olive oil sector itself, in order to ensure that producers receive fair prices that cover production costs. It is currently only profitable to produce high quality oil, but to do so there has to be an increase in demand, and in this process, the behaviour of the restaurant sector is key.

Thirdly, restaurants that seek to stand out from the crowd can find “healthy eating” as an effective strategy for market positioning and differentiation, given that consumers value health above all else in their food choices. Indeed, previous studies have demonstrated that health claims drive greater consumption of healthy foods [27,51,52,53].

On another note, it is worth contemplating that the situation brought about by the coronavirus pandemic and the ensuing economic crisis will lead to a drop in consumption outside the home. However, this observation does not alter the results of this study, i.e., that restaurants are not meeting the health demands of consumers who, whether by choice or necessity, eat outside the home. In fact, future consumers may become even more demanding as a result of the pandemic, seeking out both healthier foods and better hygiene when eating out, which would give establishments of the hostelry and restaurant sector an opportunity to stand out by promoting these features.

Summing up, we conclude that only 2% of the chefs surveyed to represent quality restaurants in Spain considered the “health” attribute to be a relevant criterion in their olive oil purchase decisions, and this attribute is not used by restaurants as an element of differentiation and positioning in the market. This fact has significant consequences: (1) The Spanish restaurant sector is not responding to consumer demand for healthy foods (in this case, olive oils). (2) This restaurant sector behaviour limits the growth potential of the demand for extra virgin olive oil, due not only to lower purchase volume but also to the “influencer” effect of restaurant chefs. Thus, the Spanish Olive Oil Interprofessional Organization and other such bodies should rethink their promotional strategy along the lines indicated above. (3) Basing the market positioning of a restaurant or café on “health” represents a sales opportunity (probably more so in post-COVID times).

On a final note, the results obtained point to future potential lines of research. Although the “health” attribute has more relative importance in the case of EVOO, in general terms, the ranking of motives and attributes is similar, both between types of oil and between uses, which may indicate a degree of confusion surrounding the subject of oils and fats. In other words, the reason why chefs give little weight to health as a relevant criterion in their buying decisions may be that they know little about the health benefits of olive oils. Thus, it would be interesting to find out whether they are able to distinguish between the different commercial categories of olive oil by correctly identifying their main characteristics.

## Figures and Tables

**Table 1:** Profile of sample.

Characteristics	Number of Respondents
	(Percentage over 400)
Gender	
Male	342 (85.5)
Female	58 (14.5)
Age group (years)	
<30	43 (10.8)
31–40	124 (31.0)
41–50	122 (30.5)
>50	111 (27.7)
Culinary training	
Experience	157 (39.2)
Academic	243 (60.8)
Quality criteria	
2–3 Michelin stars or 3 Repsol suns	13 (3.3)
1 Michelin star or 2 Repsol suns	89 (22.3)
1 Repsol sun	131 (32.8)
Average menu price > 30 €	72 (18.0)
Average menu price < 30 €	95 (23.8)

**Table 2:** Factors or reasons for purchase (% of subjects who name each criterion relative to total number of subjects who buy that type of oil, multiple answers).

Usage Setting→	Kitchen			Dining Room		
↓Criteria/Quality→	EVOO	VOO	OO	EVOO	VOO	OO
Flavour	78.44	80.80	75.62	79.73	85.60	81.59
Confidence in supplier	23.21	27.20	17.41	22.22	24.00	17.42
Price	32.67	41.60	39.80	31.04	39.20	35.33
Brand	7.19	10.40	3.99	7.19	11.20	4.49
Olive variety	13.73	13.60	12.94	13.06	15.20	9.96
Area of origin	19.28	16.80	16.92	16.34	15.20	15.43
Organic	1.64	0.80	0.00	1.96	0.80	0.00
Health	1.31	0.80	1.00	0.65	0.00	0.00
Protected Designation of Origin (PDO)	0.98	1.60	1.50	1.63	1.60	1.50
Quality certificates/awards	14.05	8.80	15.42	13.40	8.80	15.42
Other	20.91	16.0	17.91	18.95	15.20	17.91

Note: The highest quality and healthiest olive oils, listed in order, are as follows: extra virgin olive oil (EVOO), virgin olive oil (VOO), and olive oil (OO).

**Table 3:** Use of the criteria “brand” and “health” in choosing EVOO for dining room purposes (% of subjects who cite each criterion relative to total subjects interviewed in same category).

Restaurant Quality Band or Category	Brand	Health
2–3 Michelin stars or 3 Repsol suns	0.00	7.69
1 Michelin stars or 2 Repsol suns	3.37	1.12
1 Repsol sun	3.05	0.00
Menu > 30 €	8.33	0.00
Menu < 30 €	9.47	0.00

## Data Availability

Data presented in this study are available on request from the corresponding author.

## Supplementary Materials

The Supplementary Materials are available online at https://www.mdpi.com/2072-6643/13/2/445/s1. Supplementary material: Survey questionnaire.
