# Supplementary material for: Do Chefs Value Health in Their Olive Oil Purchasing Decisions?
Source: Nutrients. 2021 Jan 29;13(2):445. doi: 10.3390/nu13020445 (PMC7912701; doi:10.3390/nu13020445)
Supplement: Supplementary file 1 [file nutrients-13-00445-s001.pdf]

Supplementary material: Survey questionnaire

Consumption of vegetable oils

1. List the types of vegetable oils used according to the cooking method (multiple choice)

Cooking method/Use	Extra virgin olive oil (EVOO)	Virgin olive oil (VOO)	Olive oil (OO)
Raw (dressing, emulsion, bread, etc.)			
Sauté			
Frying pan			
Electric fryer			
Stew			
Bake			
Patisserie			
Preserves/confit			
Mayonnaise			

2. Approximately how much olive oil do you buy for your kitchen per month?

	Extra virgin olive oil	Virgin olive oil	Olive oil
Monthly amount (litres)			

Selection criteria in the purchase of olive oils

3. What factors do you consider to be most important when buying extra virgin olive oil for your kitchen? (Responses not suggested, open answer, maximum of 3)

____________________________________ Is there anything else?

____________________________________ Is there anything else?

____

4. What factors do you consider to be most important when buying extra virgin olive oil for your dining room? (Responses not suggested, open answer, maximum of 3)

____________________________________ Is there anything else?

____________________________________ Is there anything else?

____

5. What factors do you consider to be most important when buying virgin olive oil for your kitchen? (Responses not suggested, open answer, maximum of 3)

___________________________________ Is there anything else?

___________________________________ Is there anything else?

____

6. What factors do you consider to be most important when buying virgin olive oil for your dining room? (Responses not suggested, open answer, maximum of 3)

___________________________________ Is there anything else?

___________________________________ Is there anything else?

____

7. What factors do you consider to be most important when buying olive oil for your kitchen? (Responses not suggested, open answer, maximum of 3)

___________________________________ Is there anything else?

___________________________________ Is there anything else?

____

8. What factors do you consider to be most important when buying olive oil for your dining room? (Responses not suggested, open answer, maximum of 3)

___________________________________ Is there anything else?

___________________________________ Is there anything else?

____

Restaurant and interviewee descriptors

9. Characteristics of the restaurant:

- Number of Repsol suns ………………………………………………
- Number of Michelin stars ……………………………………………..
- Average menu price less than 30 € ……………………………….
- Average menu price more than 30 € …………………………….

10. Description of type of cuisine (open answer):

………
………
……..

11. Age of interviewee: …………………………

12. Gender of interviewee:

- Male
- Female

13. Culinary training of interviewee (open answer):

………
………
……..
